# 816 Modified Burn Comb Device with Increased Interspace for Accurate Measurements of Burn Wound Progression

**DOI:** 10.1093/jbcr/iraf019.347

**Published:** 2025-04-01

**Authors:** Andrea Fourcaudot, David Silliman, Brian Smith, Ping Chen, S L Rajasekhar Karna, Kai Leung

**Affiliations:** US Army Institute of Surgical Research; US Army Institute of Surgical Research; US Army Institute of Surgical Research; US Army Institute of Surgical Research; US Army Institute of Surgical Research; US Army Institute of Surgical Research

## Abstract

**Introduction:**

One complication of burn injury is burn progression, a dynamic process that continues for 48-72 hours after burn injury. The rat burn comb model developed by Regas and Ehrlich (1992), and subsequent modifications, allows for the quantification of horizontal burn conversion which influences total body surface area of a burn. This model is widely used and highly valuable for studying horizontal burn wound conversion. However, this controlled horizontal burn poses challenges in measuring the quantifiable burn progression outcomes, mainly the determination of viable tissue in the zone of stasis. Due to an overlap in the necrotic tissue (eschar), the burn progression using the traditional 4-prong device with a 5 mm interspace often results in the full conversion of the interspace within 72 hours. Modifying this comb to a 3-prong device with an increased interspace of 15 mm eliminates the eschar overlap and allows for the precise measurement of viable tissue with intact vasculature.

**Methods:**

All rats were anesthetized with isoflurane and analgesia with BuprenorphineSR was provided prior to and post all procedures. Burn injuries generated on anesthetized Sprague-Dawley rats using the 4-prong comb device traditionally cited in literature (three 5 mm interspaces) are compared with burn injuries from the modified 3-prong comb (two 15 mm interspaces). Using ImageJ, digital images of the burns obtained on day 0 immediately after burn and days 1, 2, 3, and 7 post-burn were measured for changes in the necrotic zone of coagulation, indicative of changes of burn wound size. Tissue was harvested post-euthanasia on day 7 for histopathological analysis which included measurements and scoring of inflammatory cell infiltration, vascular occlusion, and extent of tissue damage by a board-certified histopathologist.

**Results:**

Both comb devices generated full-thickness burns at 20 seconds contact time and digital images of burns with the 3-prong device showed no eschar overlap in the interspace as compared to the 4-prong device. Histopathological analysis in the increased interspace model showed an increase in distance between the eschar and larger areas of viable tissue. A zone of hyperemia with intact and healthy vasculature was also observed.

**Conclusions:**

Using the modified comb burn device, we were able to accurately determine the zone of stasis and measure viable tissue in the interspace without overlap in zones of coagulation or stasis. This highly measurable zone of stasis lends itself to more consistent pathophysiology scoring and will aid in the interpretation of treatment effects in future studies.

**Applicability of Research to Practice:**

Modified burn comb device can be used for testing therapeutics to reduce burn progression.

**Funding for the Study:**

Studies are supported by the Congressional Military Dental Research Program administered by the Naval Medical Research Command Naval Advanced Medical Development.

